# The association between childhood trauma and tobacco smoking in patients with psychosis, unaffected siblings, and healthy controls

**DOI:** 10.1007/s00406-023-01754-z

**Published:** 2024-01-17

**Authors:** Justine de With, Heleen S. van der Heijden, Therese van Amelsvoort, Maud Daemen, Claudia Simons, Behrooz Alizadeh, Daphne van Aalst, Lieuwe de Haan, Jentien Vermeulen, Frederike Schirmbeck

**Affiliations:** 1 Department of Psychiatry Amsterdam, UMC (Location AMC), Meibergdreef 9, 1105 AZ Amsterdam, The Netherlands; 2 Department of Psychiatry and Neuropsychology, School for Mental Health and Neuroscience, Maastricht University Medical Center, Maastricht, The Netherlands; 3 GGzE Institute for Mental Health Care, Eindhoven, The Netherlands; 4 Department of Psychiatry, Rijksuniversiteit Groningen, University Medical Center Groningen, Groningen, The Netherlands; 5 Department of Epidemiology, University Medical Center Groningen, Groningen, The Netherlands; 6 Arkin, Institute for Mental Health, Amsterdam, The Netherlands; 7 Department of Public Mental Health, Central Institute of Mental Health, Medical Faculty Mannheim, Heidelberg University, Mannheim, Germany

**Keywords:** Psychosis, Childhood trauma, Tobacco, Smoking

## Abstract

In patients with psychosis, rates of tobacco smoking and childhood trauma are significantly higher compared to the general population. Childhood trauma has been proposed as a risk factor for tobacco smoking. However, little is known about the relationship between childhood trauma and smoking in psychosis. In a subsample of the Genetic Risk and Outcome of Psychosis study (760 patients with psychosis, 991 unaffected siblings, and 491 healthy controls), tobacco smoking was assessed using the Composite International Diagnostic Interview and childhood trauma was measured with the Childhood Trauma Questionnaire. Logistic regression models were used to assess associations between trauma and smoking, while correcting for confounders. Positive associations were found between total trauma, abuse, and neglect, and an increased risk for smoking in patients, while correcting for age and gender (OR_trauma_ 1.77, 95% CI 1.30–2.42, *p* < 0.001; OR_abuse_ 1.69, 95% CI 1.23–2.31, *p* = 0.001; OR_neglect_ 1.48, 95% CI 1.08–2.02, *p* = 0.014). In controls, total trauma and abuse were positively associated with smoking, while correcting for age and gender (OR_trauma_ 2.40, 95% CI 1.49–3.88, *p* < 0.001; OR_abuse_ 2.02, 96% CI 1.23–3.32, *p* = 0.006). All associations lost their significance after controlling for additional covariates and multiple testing. Findings suggest that the association between childhood trauma and tobacco smoking can be mainly explained by confounders (gender, cannabis use, and education) in patients with psychosis. These identified aspects should be acknowledged in tobacco cessation programs.

**Supplementary Information:**

The online version contains supplementary material available at 10.1007/s00406-023-01754-z.

## Introduction

In patients with psychosis, the prevalence of tobacco smoking is three times higher compared to the general population [1, 2]. Several (non-mutually exclusive) theories exist to explain this extremely high smoking prevalence in psychosis. Among them are the shared vulnerability hypothesis [3] and the hypothesis that the smoking–psychosis relationship is confounded by other risk factors for psychosis, such as childhood trauma [4, 5].

A previous study showed that children who experienced childhood trauma were almost three times more likely to have psychotic symptoms at the age of 18 years [6]. Furthermore, depression and anxiety and rates of suicidality have been shown to be higher in patients with psychosis exposed to childhood trauma compared to patients who did not experience trauma [7]. However, the impact of childhood trauma on other outcomes in patients with psychosis is not well established. Considering the fact that trauma is highly associated with tobacco smoking in the general population [8], it is of interest to investigate the relationship between trauma and smoking in patients with psychosis in order to (partially) explain the high smoking prevalence among psychosis. This might also have clinical relevance, since smoking and trauma are associated with worse clinical outcomes in these patients [9, 10].

The relationship between trauma and substance use in psychosis has reached research interest over the last decades. In a recent systematic review [11], the majority of included studies (16 out of 19) found a direct relationship between trauma and use of different substances. However, only one of the included studies explored associations with tobacco smoking [12], which found no relationship between exposure to childhood trauma and tobacco smoking. In another study, Rey and colleagues (2017) reported that the experience of childhood trauma was associated with severe nicotine dependence in patients with psychosis, compared to mild nicotine dependence [13]. By contrast, in a partly overlapping patient sample, Mallet and colleagues [14] did not find an association between childhood trauma and current tobacco smoking in patients with psychosis.

In summary, the small number of studies that have been conducted on the association between childhood trauma and tobacco smoking in patients with psychosis has shown inconsistent findings [12–14], whereas trauma has consistently been identified as risk factor for smoking in healthy controls [15]. Moreover, only one study investigated the association between distinct types of childhood trauma and tobacco smoking [12]. To our knowledge, this is the first study investigating the relationship between childhood trauma and tobacco smoking in a sample of patients with a non-affective psychosis, unaffected siblings, and healthy controls. By including unaffected siblings, we were able to investigate associations in participants with an increased liability for psychosis, without the confounding effects of illness-related (clinical) factors. The aim was to evaluate whether experienced childhood trauma was associated with tobacco smoking across groups. We hypothesized that the experience of childhood trauma would be associated with an increased risk of tobacco smoking in non-affective patients, unaffected siblings, and healthy controls.

## Method

### Study design and participants

This study was performed within the Genetic Risk and Outcome of Psychosis (GROUP) study. GROUP is a Dutch multi-site naturalistic follow-up study (after 3 and 6 years) designed to investigate risk and protective factors influencing the onset and course of psychotic disorders in patients, their unaffected family members, and non-related controls. Patients were recruited in selected representative geographical areas of the Netherlands, including Amsterdam, Groningen, Maastricht, and Utrecht, and 36 mental health care institutes, and (the Dutch-speaking part of) Belgium. The study took place from April 2004 to December 2013. Patients were identified through clinicians, whose caseload was screened for inclusion criteria. Patients presenting at these services either as outpatients or inpatients were recruited. Patients identified as potentially eligible were asked for informed consent for participation in the study and for contacting their first-degree siblings. Random mailings to addresses were used to recruit controls in the selected areas. Assessments took place at one of the study sites. If participants were unable to visit the study site, in-home assessments were being offered. Interviewers received extensive training to optimize the reliability of measurements [16].

Inclusion criteria for all groups were an age range from 16 to 50 years, able and willing to give written informed consent, and proficiency in the Dutch language. Moreover, patients had to meet the DSM-IV-TR criteria for a non-affective psychotic disorder, assessed with the Comprehensive Assessment of Symptoms and History (CASH) [17] or the Schedules for Clinical Assessment in Neuropsychiatry (SCAN) [18]. Exclusion criteria for healthy controls and siblings were a lifetime psychotic disorder or a first-degree family member with a lifetime psychotic disorder.

The present study consisted of a subsample of 760 patients, 991 siblings, and 491 controls of whom complete datasets for childhood trauma and tobacco smoking were available at baseline (Maastricht) or 3 year follow-up (the other research sites) (Supplement 1 and 2). The Medical Ethics Committee of the Academic Medical Centre of Utrecht approved the study (approval number 04/003-O). Written informed consent was obtained from participants before they were enrolled in the study. GROUP data release 8.0 was used for current analyses.

### Measurements

#### Tobacco smoking

The Dutch version of the Composite International Diagnostic Interview (CIDI) was used to assess the quality and severity of tobacco smoking [19, 20]. We collected CIDI data from the baseline assessment in Maastricht and from the 3-year follow-up assessment in Amsterdam, Utrecht, and Groningen, corresponding with the available childhood trauma data. Participants were defined as “smokers” if they had used tobacco daily for at least 1 month in the past 12 months. Furthermore, tobacco smoking patients were asked how many cigarettes they smoked per day in the period of most severe smoking. High acceptability and reliability has previously been demonstrated for the CIDI [21].

#### Childhood trauma

The Dutch Childhood Trauma Questionnaire (CTQ) was used to assess experienced childhood trauma [22]. We collected CTQ data from the baseline assessment in Maastricht and from the 3-year follow-up assessment in Amsterdam, Utrecht, and Groningen, based on availability. The CTQ is a 25-item self-report questionnaire and measures physical abuse and neglect, sexual abuse, and emotional abuse and neglect before the age of 18. Items are rated on a 5-point Likert rating scale ranging from one (never true) to five (very often true). The CTQ is observed to be an adequate measurement with good criterion-related validity and reliability, and with high internal consistency ranging from 0.79 to 0.94 [23, 24]. For all analyses, the total trauma scale as well as subscale scores for abuse and neglect were used (mean score of all items combined). In line with previous work, the trauma scores were dichotomized into high and low trauma. The cut-off was defined as the 80th percentile of scores for the healthy controls [10]. The cut-offs for the total trauma rating, abuse rating, and neglect rating were therefore set at 1.52, 1.33, and 1.90, respectively.

#### Covariates

Based on their putative association with childhood trauma and/or tobacco smoking, the following a priori covariates were selected: age, gender, years of education, severity of the psychopathological dimensions (positive symptoms, negative symptoms, and depressive symptoms), and cannabis use [9, 25, 26]. In patients, the use of antipsychotic medication (yes/no/unknown) was also added as a covariate [13]. We classified antipsychotics as first- or second-generation antipsychotics. Current dosage of antipsychotic medication was converted into chlorpromazine equivalents (CPZE), meaning that the current dose was equivalent to 100 mg of oral dose of chlorpromazine. Sociodemographic data were evaluated using a self-reported questionnaire specifically developed for the GROUP-study. Cannabis use was evaluated with urine analysis (positive or negative) at all assessments (cut-off 50 ng/ml). The presence of (sub)clinical psychotic experiences in patients, siblings, and controls was measured with the Community Assessment of Psychotic Experiences (CAPE) [27]. This 42-item self-report questionnaire assesses the prevalence of positive, negative, and depressive symptoms over lifetime (baseline assessment) or the past 3 years [3-year follow-up assessment]. Each of the items was rated in terms of frequency on a scale from zero (absent) to three (almost always). A mean total score was calculated for the different subscales, with a higher score reflecting a higher frequency of symptoms. In patients, the severity of psychotic symptoms was also assessed with the Positive and Negative Syndrome Scale (PANSS) [28]. This 30-item clinician-rated interview assesses positive symptoms, negative symptoms, and general psychopathology. Each of the items was rated on a seven-point scale in terms of severity on a scale from zero (absent) to seven (extreme). A mean total score was calculated for the different subscales, with a higher score reflecting a higher severity of symptoms.

### Statistical analyses

All statistical analyses were performed using Statistical Package for the Social Sciences (IBM SPSS Statistics) version 28.0. Normality was checked visually for all numerical variables with probability plots and scatterplots of standardized residuals. Differences between groups regarding baseline demographic and clinical characteristics were compared by one-way (welch) ANOVA and Chi^2^ test, for nominal and categorical data, respectively. Hierarchical logistic regression models were used to examine whether childhood trauma subscales (binary independent variables) were associated with tobacco smoking (binary dependent variable), whilst correcting for age and gender (model 1: age and gender entered at step 1; trauma subscales entered at step 2). If the model showed significant results, these analyses were repeated with models including all additional covariates (model 2: age and gender entered at step 1; education, positive symptoms, negative symptoms, depressive symptoms, and cannabis use entered at step 2; trauma subscales entered at step 3).

To test significant findings in model 1 and 2, we replaced the substance use status by the numeric outcome variable number of cigarettes, again accounting for the aforementioned covariates. In the patient group, analyses were first repeated with antipsychotic medication as a covariate to the previously mentioned set and PANSS scores were used instead of CAPE scores. Second, we added the type of antipsychotic (first or second generation) as well as the dose (equivalent dose chlorpromazine) to our logistic regression model. All statistical tests were two-sided and statistical significance was set at *p* < 0.017 (0.05/3) according to Bonferroni multiple testing correction.

## Results

In Table 1, sociodemographic and clinical characteristics are represented for all patients, siblings, and controls. Differences between groups regarding baseline demographic and clinical characteristics are shown in Supplement 3. See Supplement 4 for further detailed information on comorbid substance use and Supplement 5 on type and dose of antipsychotic medication use.

**Table 1:** Study sample characteristics of included patients, siblings, and controls

	Patients (*N* = 760)	Siblings (*N* = 991)	Controls (*N* = 491)
Gender (male)	569 (74.9%)	431 (43.5%)	217 (44.2%)
Age (in years)	27.6 (7.4)	32.8 (13.0)	31.0 (10.8)
Education (in years)	12.8 (3.7)	13.4 (3.8)	14.6 (3.3)
Smokers (yes)	491 (64.6%)	326 (32.9%)	115 (23.4%)
If yes: number of cig/day	19.1 (9.4)	13.1 (8.6)	11.7 (7.7)
Total trauma			
High	366 (48.2%)	290 (29.3%)	107 (21.8%)
Low	394 (51.8%)	701 (70.7%)	384 (78.2%)
Abuse trauma			
High	342 (45.0%)	255 (25.7%)	98 (20.0%)
Low	418 (55.0%)	736 (74.3%)	393 (80.0%)
Neglect trauma			
High	340 (44.7%)	303 (30.6%)	113 (23.0%)
Low	420 (55.3%)	688 (69.4%)	378 (77.0%)
PANSS			
Positive subscale	11.6 (4.9)		
Negative subscale	12.0 (5.3)		
General subscale	25.0 (7.4)		
CAPE			
Positive symptoms	0.6 (0.5)	0.1 (0.2)	0.1 (0.1)
Negative symptoms	1.0 (0.5)	0.5 (0.4)	0.4 (0.3)
Depressive symptoms	0.9 (0.6)	0.6 (0.4)	0.5 (0.4)
Cannabis use			
Yes	88 (11.6%)	49 (4.9%)	17 (3.5%)
No	569 (74.9%)	694 (70.0%)	442 (90.0%)
Unknown	103 (13.6%)	248 (25.0%)	32 (6.5%)
DSM IV-TR diagnosis			
Schizophrenia	526 (69.2%)	–	–
Schizoaffective disorder	87 (11.4%)	–	–
Unspecified psychosis	92 (12.1%)	–	–
Other diagnosis	15 (2.0%)	–	–
Illness duration (in years)	7.0 (4.6)	–	–
Antipsychotic medication^a^		–	–
Yes	653 (85.5%)	–	–
No	4 (0.5%)	–	–
Unknown	107 (14.0%)	–	–

Data are *n* [indicated by (%)] or mean (SD)

*Cig* cigarettes, *DSM IV-TR*  Diagnostic and Statistical Manual of Mental Disorders version IV-TR, *CAPE* Community Assessment of Psychic Experience, frequency subscales, *PANSS* positive and Negative Syndrome Scale

^a^See supplement 5 for more detailed information regarding the type and dose of antipsychotics

In patients, all trauma scales were significantly associated with tobacco smoking while controlling for age and gender (OR_trauma_ 1.77, 95% CI 1.30–2.42, *p* < 0.001; OR_abuse_ 1.69, 95% CI 1.23–2.31, *p* = 0.001; OR_neglect_ 1.48, 95% CI 1.08–2.02, *p* = 0.014) (Table 2). Detailed information on the confounding effects of age and gender are shown in Supplement 6. As a sensitivity analysis, we replaced smoking status as outcome variable with the number of cigarettes (Supplement 7). Total trauma and abuse were significantly associated with the number of cigarettes smoked in patients when correcting for age and gender. For neglect, however, the association lost its significance. All associations lost significance after adding adding severity of the psychopathological dimensions (positive symptoms, negative symptoms, and depressive symptoms), cannabis use, and education as confounders to the models (Table 2). Detailed information on the confounding effects of all covariates is shown in Supplement 8. Findings were not affected when repeating analyses of model 2 by adding antipsychotic medication use and PANSS scores instead of CAPE scores (Supplement 9) nor when antipsychotic medication dosage and type were added (Supplement 10).

**Table 2:** Associations between childhood trauma and tobacco smoking by group status while correcting for confounders

	Patients		Siblings		Controls	
	Model 1^1^ (*N* = 760)	Model 2^2^ (*N* = 610)	Model 1^1^ (*N* = 991)	Model 2^2^ (*N* = 718)	Model 1^1^ (*N* = 491)	Model 2^2^ (*N* = 435)
Trauma	OR [95% CI]	OR [95% CI]	OR [95% CI]	OR [95% CI]	OR [95% CI]	OR [95% CI]
Total	1.77 [1.30–2.42]*	1.32 [0.91–1.91]	1.37 [1.02–1.84]	–	2.40 [1.49–3.88]*	1.78 [1.00–3.14]
Abuse	1.69 [1.23–2.31]*	1.35 [0.92–1.98]	1.11 [0.81–1.50]	–	2.02 [1.23–3.32]*	1.41 [0.77–2.56]
Neglect	1.48 [1.08–2.02]*	1.11 [0.77–1.60]	1.15 [0.86–1.55]	–	1.59 [0.98–2.57]	–

*OR*  Odds Ratio, *95% CI*  95% confidence interval

^1^Model 1: corrected for age and gender

^2^Model 2: corrected for age, gender, education, positive symptoms, negative symptoms, depressive symptoms, and cannabis use

*p value < 0.017, considering Bonferroni correction for multiple testing

In siblings, none of the trauma scales were significantly associated with tobacco smoking in model 1 (confounders: age and gender) while correcting for multiple testing (Table 2). The confounding effects of age and gender are shown in Supplement 6.

In controls, total trauma and abuse were associated with tobacco smoking while controlling for age and gender (OR_trauma_ 2.40, 95% CI 1.49–3.88, *p* < 0.001; OR_abuse_ 2.02, 96% CI 1.23–3.32, *p* = 0.006) (Table 2). Detailed information on the confounding effects of age and gender is shown in Supplement 6. When replacing smoking status as outcome variable with the number of cigarettes, results were not significant affected (Supplement 11). After adding the additional covariates, the associations lost significance (OR 1.78, 95% CI 1.00–3.14, *p* = 0.048) (Table 2). Detailed information on the confounding effects of all covariates is shown in Supplement 8.

## Discussion

### Summary of main findings

The present study explored the associations between different modalities of childhood trauma (total, abuse, and neglect) and tobacco smoking in a sample of patients with a non-affective psychotic disorder, unaffected siblings, and healthy controls. The positive associations that we found in patients between childhood trauma and tobacco smoking while correcting for age and gender lost significance after controlling for severity of the psychopathological dimensions (positive symptoms, negative symptoms, and depressive symptoms), cannabis use, and educational level. Although, also in the fully corrected model, there is a positive association (albeit non-significant). We found that childhood trauma and abuse increased the risk of tobacco smoking in controls, but again significance was lost after controlling for confounders. Concerning siblings, none of the trauma scales were significantly associated with tobacco smoking.

### Previous literature

To the best of our knowledge, this was the first study investigating the relationship between childhood trauma and tobacco smoking in a sample of patients with psychosis, unaffected siblings, and healthy controls. In line with our findings in the patient group, Mallet et al. [14] also did not find an association between trauma and smoking in patients with psychosis (*N* = 474). Rey et al. [13] found that the experience of childhood trauma was associated with severe nicotine dependence compared to mild nicotine dependence (OR 1.03). Our result of an association between trauma and the number of cigarettes smoked per day (as sensitivity analysis) builds upon this previous study; further studies should validate the direction of this association. Noteworthy, in the study of Rey et al., this association was independent of other confounding variables including severity of symptomatology. In the previous literature, smoking in psychosis was found to be associated with positive, negative, and depressive symptoms [9]. One could speculate that heavy smokers are a particularly vulnerable group who may be underrepresented in our sample, which can explain the independence of positive, negative, and depressive symptoms in the association between childhood trauma and tobacco smoking.

Interestingly, in the patient group, gender played a confounding role on the association between trauma and tobacco smoking in the current study, while this was not found in healthy controls. Our finding in patients is in line with prior psychosis studies reporting higher rates of tobacco smoking in men compared to women [13]. Combining this with the previously demonstrated higher frequency of childhood trauma in women with psychosis compared to men [29], it is interesting for future studies to explore the differential impact of gender on the association between trauma and tobacco smoking.

In the current study, cannabis was the most contributing confounder when investigating the association between trauma and tobacco smoking. It has, indeed, been found that the use of cannabis and tobacco smoking are highly correlated in psychosis [30]. Furthermore, in the previous psychosis literature, the link between trauma and cannabis use has been well established [11]. It is suggested that cannabis is used in an attempt to cope with the emotional symptoms of childhood trauma [31, 32]. The link between trauma and cannabis use has also been evaluated as an environmental risk factor for developing psychosis. One study found that these two have a greater than additive interaction in increasing the risk of psychosis [33]. Future studies should investigate this possible interaction for tobacco smoking as well.

Regarding the role of education on the relationship between trauma and smoking in the current study, it has indeed been found that smoking patients with psychosis have lower levels of education compared to their non-smoking peers [9]. With respect to the relationship between trauma and educational level, it is suggested that lower childhood socio-economic status is associated with an increased risk of experiencing childhood trauma in the general population [34]. Vice versa, childhood trauma is prospectively associated with lower educational levels [35]. These findings highlight the importance of education in both signaling and treatment of childhood trauma.

Against our expectations, we did not find significant associations between childhood trauma and tobacco smoking in unaffected siblings. To the best of our knowledge, no studies are performed with a similar sample. Including a subgroup of siblings with similar predispositions as patients could provide information about the role of illness-related factors in the link between smoking and trauma. Our findings suggest that childhood trauma has a stronger bearing on tobacco smoking in patients and controls, while in siblings other factors might play a more important role. Our findings suggest that childhood trauma has a stronger bearing on tobacco smoking in patients and controls, while in siblings, other (psychological) factors might play a more important role. Future studies should explore the relationship between participants with different vulnerabilities for psychosis.

With respect to our findings in healthy controls, the association between trauma and abuse did not survive correction for multiple testing. When comparing our results with the general population, research on the impact of childhood trauma has clearly demonstrated a link between trauma and tobacco smoking [8, 15]. As the group of controls in our study was relatively small, we might have missed smaller effects, due to a lack of power.

### Clinical implications

Our study adds to the body of literature by underlining the importance of confounders in the relationship between smoking and trauma. Several, non-mutually exclusive mechanisms could play a role in the relationship between childhood trauma and tobacco smoking in psychosis. From a psychological point of view, the experience of childhood trauma is highly associated with psychological distress, negative affect, and stress [36]. It might be that patients with psychosis use tobacco smoking to cope with the negative effects of trauma exposure. Indeed, a recent study found that maladaptive coping influenced the relationship between life events and the risk of smoking [37].

Attachment style might also play a part in the relationship between childhood trauma and tobacco smoking, as was previously shown in a adolescents at risk for aggressive behaviour [38]. Interestingly, a recent study found that the relationship between insecure-anxious attachment and problematic substance use was confirmed and found to be fully mediated by dysfunctional coping [39]. However, it was unclear whether tobacco use was included in this study.

From a biological point of view, the increased prevalence of childhood trauma and smoking in patients with psychosis could be associated with several adaptions caused by both smoking and the experience of childhood trauma that make the brain more vulnerable for psychosis. For example, trauma has been associated with changes in the endocrine system, immune system, and brain structure [40]. Tobacco smoking is also associated with oxidative stress, inflammation, and white matter lesion progression [41]. It is suggested that tobacco smoking might be a ‘second hit’ and further disrupt systems associated with psychosis. Furthermore, shared environmental risk factors for psychosis as cannabis use [42], tobacco smoking [43], and trauma exposure [25] seem to be involved in a complex relationship with each other according to the current study.

In the present study, we focused on the risk of tobacco use in trauma-exposed patient while controlling for cannabis use. We did not assess substance use disorders, nor did we investigate the influence of dual disorders involving cannabis use (or alcohol and other drugs) on the association between childhood trauma and tobacco smoking. Dual disorders (i.e., schizophrenia and SUD) are associated with worse mental health compared to patients with schizophrenia alone [44]. Future studies could explore the associations between trauma and patients with dual disorders, especially considering that tobacco smoking is highly associated with the risk of cannabis use [45].

### Strengths and limitations

Strengths of this study are the relatively large sample size, and the inclusion of patients, siblings, and controls to investigate associations in participants with an increased psychosis liability without the confounding effects of illness-related (clinical) factors. However, the current study has several limitations. First, retrospective design of the study does not allow for any conclusions regarding causation and introduced the risk of a recall bias regarding the experienced childhood trauma. In addition, self-report questionnaires (CTQ) have been used to assess experienced childhood trauma and tobacco smoking, which introduces the possibility of self-report biases. Moreover, the CTQ does not include detailed information about the experienced trauma, such as the duration of the experience and the age of occurrence. Furthermore, the CTQ does not include other types of childhood trauma, such as bullying or loss of a parent. Third, we defined participants as smokers if they had used tobacco daily for at least 1 month in the past 12 months. We were not able to include the age of onset of tobacco smoking. Future studies with more detailed assessment of smoking behaviour are needed to elucidate the effect of both smoking frequency, severity, and age of onset.

Fourth, while our findings indicate that the use of antipsychotic medication did not alter the relationship between experienced trauma and tobacco smoking, it is important to interpret this result with the awareness that more detailed information on duration and compliance is lacking. Fifth, patients in the GROUP study sample are relatively high functioning with a probably lower severity of symptoms. This warrants cautiousness in generalization of our findings. Finally, the sample size of the healthy control group was relatively small. Including a larger sample size would be of value to increase power.

### Further research

Our findings imply when treating patients with psychosis for trauma-related symptoms and/or tobacco addiction, one should focus factors as well, for example comorbid use of cannabis.

Future research could explore the role of attachment and coping styles on the relationship between childhood trauma and tobacco smoking in patients with psychotic disorders and unaffected siblings.

## Supplementary Information

Below is the link to the electronic supplementary material.

Supplementary file1 (DOCX 40 KB)
