# Correction: The association between childhood trauma and tobacco smoking in patients with psychosis, unaffected siblings, and healthy controls

**DOI:** 10.1007/s00406-024-01823-x

**Published:** 2024-05-16

**Authors:** Justine de With, Heleen S. van der Heijden, Therese van Amelsvoort, Maud Daemen, Claudia Simons, Behrooz Alizadeh, Daphne van Aalst, Lieuwe de Haan, Jentien Vermeulen, Frederike Schirmbeck

**Affiliations:** 1 Department of Psychiatry Amsterdam, UMC (Location AMC), Meibergdreef 9, 1105 AZ Amsterdam, The Netherlands; 2 Department of Psychiatry and Neuropsychology, School for Mental Health and Neuroscience, Maastricht University Medical Center, Maastricht, The Netherlands; 3 GGzE Institute for Mental Health Care, Eindhoven, The Netherlands; 4 Department of Psychiatry, Rijksuniversiteit Groningen, University Medical Center Groningen, Groningen, The Netherlands; 5 Department of Epidemiology, University Medical Center Groningen, Groningen, The Netherlands; 6 Arkin, Institute for Mental Health, Amsterdam, The Netherlands; 7 Department of Public Mental Health, Central Institute of Mental Health, Medical Faculty Mannheim, Heidelberg University, Mannheim, Germany

**Correction: European Archives of Psychiatry and Clinical Neuroscience** 10.1007/s00406-023-01754-z

In this article the author’s name Heleen S. van der Heijden was incorrectly written as Sanne van der Heijden.

The original article has been corrected.

