# Supplementary material for: The association between childhood trauma and tobacco smoking in patients with psychosis, unaffected siblings, and healthy controls
Source: Eur Arch Psychiatry Clin Neurosci. 2024 Jan 17;274(7):1575–83. doi: 10.1007/s00406-023-01754-z (PMC11422427; doi:10.1007/s00406-023-01754-z)
Supplement: Supplementary file 1 — Supplementary file1 (DOCX 40 KB) [file 406_2023_1754_MOESM1_ESM.docx]

**Supplementary Appendix**

This appendix is supplement to:

*The association between childhood trauma and tobacco smoking in patients with psychosis, unaffected siblings, and healthy controls*

**Index**:

**Supplement 1.** Participants with complete data on childhood trauma and smoking by assessment period and by site

**Supplement 2.** Participants with missing data on covariates.

**Supplement 3** Comparison of study sample characteristics of included patients, siblings and controls.

**Supplement 4.** Frequencies of comorbid substance use (tobacco smoking and cannabis use) by group status

**Supplement 5.** Detailed information regarding the type and dose of antipsychotics

**Supplement 6.** Associations between childhood trauma and tobacco smoking by group status while correcting for age and gender (model 1)

**Supplement 7.** Associations between childhood trauma and numbers of cigarettes in patients

**Supplement 8.** Associations between childhood trauma and tobacco smoking in patients and controls while corrected for multiple confounders (model 2)

**Supplement 9.** Associations between childhood trauma and tobacco smoking in patients with additional covariates (antipsychotic medication use and PANSS scores)

**Supplement 10.** Associations between childhood trauma and tobacco smoking in patients with additional covariates (antipsychotic medication dosage, antipsychotic medication type, and PANSS scores)

**Supplement 11.** Associations between childhood trauma and numbers of cigarettes in controls

**Supplement 1.** Participants with complete data on childhood trauma and smoking by assessment period and by site

|  | *Baseline* | *3-year follow up* |
| --- | --- | --- |
| **Amsterdam** | - | 408 |
| **Groningen** | - | 449 |
| **Maastricht** | 969 | - |
| **Utrecht** | - | 416 |
| **Total** | 969 | 1273 |
| Data in N. | | |

**Supplement 2.** Participants with missing data on covariates

| **Variable** | **Patients** | **Siblings** | **Controls** |
| --- | --- | --- | --- |
| Gender | 0 (0.0%) | 0 (0.0%) | 0 (0.0%) |
| Age | 0 (0.0%) | 0 (0.0%) | 0 (0.0%) |
| Education in years | 30 (3.9%) | 18 (1.8%) | 14 (2.9%) |
| CAPE | 34 (4.5%) | 20 (2.0%) | 12 (2.4%) |
| Cannabis use | 103 (13.6%) | 248 (25.0%) | 32 (6.5%) |
| PANSS | 20 (2.6%) | - | - |
| Illness duration | 30 (3.9%) | - | - |
| Antipsychotic medication | 147 (19.3%) | - | - |
| N (%) = missing at baseline. Abbreviations: CAPE = Community Assessment of Psychic Experience, frequency subscales; PANSS=Positive and Negative Syndrome Scale. | | | |

**Supplement 3.** Comparison of study sample characteristics of included patients, siblings and controls

|  | **Patients (N=760)** | **Siblings (N=991)** | **Controls (N=491)** | **p-value** | **Post-hoc** |
| --- | --- | --- | --- | --- | --- |
| Gender (male) | 569 (74.9%) | 431 (43.5%) | 217 (44.2%) | <0.001 | Pt > Sib & Con |
| Age (in years) | 27.6 (7.4) | 32.8 (13.0) | 31.0 (10.8) | <0.001 | Pt < Con < Sib |
| Education (in years) | 12.8 (3.7) | 13.4 (3.8) | 14.6 (3.3) | <0.001 | Pt < Sib < Con |
| Smokers (yes)  If yes: number of cig/day | 491 (64.6%)  19.1 (9.4) | 326 (32.9%)  13.1 (8.6) | 115 (23.4%)  11.7 (7.7) | <0.001  <0.001 | Pt > Sib > Con |
| Total trauma |  |  |  | <0.001 | Pt > Sib > Con |
| High | 366 (48.2%) | 290 (29.3%) | 107 (21.8%) |  |  |
| Low | 394 (51.8%) | 701 (70.7%) | 384 (78.2%) |  |  |
| Abuse trauma |  |  |  | <0.001 | Pt > Sib > Con |
| High | 342 (45.0%) | 255 (25.7%) | 98 (20.0%) |  |  |
| Low | 418 (55.0%) | 736 (74.3%) | 393 (80.0%) |  |  |
| Neglect trauma |  |  |  | <0.001 | Pt > Sib > Con |
| High | 340 (44.7%) | 303 (30.6%) | 113 (23.0%) |  |  |
| Low | 420 (55.3%) | 688 (69.4%) | 378 (77.0%) |  |  |
| CAPE |  |  |  |  |  |
| Positive symptoms | 0.6 (0.5) | 0.1 (0.2) | 0.1 (0.1) | <0.001 | Pt > Sib & Con |
| Negative symptoms | 1.0 (0.5) | 0.5 (0.4) | 0.4 (0.3) | <0.001 | Pt > Sib & Con |
| Depressive symptoms | 0.9 (0.6) | 0.6 (0.4) | 0.5 (0.4) | <0.001 | Pt > Sib & Con |
| Cannabis use |  |  |  | <0.001 | Pt > Sib > Con |
| Yes | 88 (11.6%) | 49 (4.9%) | 17 (3.5%) |  |  |
| No | 569 (74.9%) | 694 (70.0%) | 442 (90.0%) |  |  |
| Unknown | 103 (13.6%) | 244 (24.4%) | 26 (5.3%) |  |  |
| Data are n (indicated by (%)) or mean (SD). Abbreviations: cig=cigarettes; DSM IV-TR= Diagnostic and Statistical Manual of Mental Disorders version IV-TR; CAPE = Community Assessment of Psychic Experience, frequency subscales; PANSS=Positive and Negative Syndrome Scale. | | | | | |

**Supplement 4.** Frequencies of comorbid substance use (tobacco smoking and cannabis use) by group status

|  | **Patients (N=760)** | **Siblings (N=991)** | **Controls (N=491)** |
| --- | --- | --- | --- |
| 0 substances | 233 (30.7%) | 480 (48.4%) | 347 (70.7%) |
| 1 substance: tobacco | 336 (44.2%) | 214 (21.6%) | 95 (19.3%) |
| 1 substance: cannabis | 3 (0.4%) | 10 (1.0%) | 3 (0.6%) |
| 2 substances | 85 (11.2%) | 39 (3.9%) | 14 (2.9%) |
| Unknown* | 103 (13.6%) | 248 (25.1%) | 32 (6.5%) |
| Data are n (%). * dual substance use unknown because of missing cannabis data. | | | |

**Supplement 5.** Detailed information regarding the type and dose of antipsychotics

| Antipsychotics | Patients (N=760) |
| --- | --- |
| Use of antipsychotics:  Yes  First generation AP  Second generation AP  Unknown type AP  Antipsychotic daily dosage (CPZE)  No  Unknown | 593 (78.0%)  61 (8.0%)  465 (61.2%)  3 (0.4%)  373.6 (420.1)*  20 (2.6%)  147 (19.3%) |
| Data in N (%) or mean (SD). Abbreviations: AP = antipsychotics; CPZE = Chlorpromazine equivalent in mg/day. *Of the 593 patients who used AP, 526 had data available regarding the dose of AP. | |

Supplement 5 (continued)

| Type of used antipsychotic medication in patients (N=529) | | |
| --- | --- | --- |
| Olanzapine  Risperidon  Clozapine  Aripiprazol  Quetiapine  Haloperidol  Pimozide  Flupentixol  Penfluridol  Zuclopentixol  Broomperidol  Paliperidon  Sulpiride  Perfenazine  Pipamperon  Unknown | 121 (22.9%)  121 (22.9%)  107 (20.2%)  57 (10.8%)  52 (9.8%)  21 (4.0%)  12 (2.3%)  6 (1.1%)  6 (1.1%)  6 (1.1%)  4 (0.8%)  3 (0.6%)  1 (0.2%)  1 (0.2%)  1 (0.2%)  3 (0.6%) | S  S  S  S  S  F  F  F  F  F  F  F  F  F  F  n.a. |
| Abbreviations: AP = antipsychotic medication. F = First generation AP. S = Second generation AP. | | |

**Supplement 6.** Associations between childhood trauma and tobacco smoking by group status while correcting for age and gender (model 1)

|  | **Patients (N=760)** | | **Siblings (N=991)** | | **Controls (N=491)** | |
| --- | --- | --- | --- | --- | --- | --- |
| *Total trauma* | OR [95% CI] | p | OR [95% CI] | p | OR [95% CI] | p |
| Age | 0.99 [0.97 – 1.01] | 0.194 | 0.98 [0.97 – 0.99] | 0.002* | 0.99 [0.97 – 1.01] | 0.199 |
| Gender | 0.43 [0.31 – 0.60] | <0.001* | 0.80 [0.61 – 1.05] | 0.101 | 0.55 [0.35 – 0.84] | 0.006* |
| Total trauma | 1.77 [1.30 – 2.42] | <0.001* | 1.37 [1.02 – 1.84] | 0.036 | 2.40 [1.49 – 3.88] | <0.001* |
|  |  |  |  |  |  |  |
| *Trauma: abuse* | OR [95% CI] | p | O R [95% CI] | p | OR [95% CI] | p |
| Age | 0.99 [0.97 – 1.01] | 0.276 | 0.99 [0.97 – 1.00] | 0.004* | 0.99 [0.97 – 1.01] | 0.265 |
| Gender | 0.42 [0.30 – 0.59] | <0.001* | 0.81 [0.62 – 1.06] | 0.131 | 0.54 [0.35 – 0.83] | 0.005* |
| Abuse | 1.69 [1.23 – 2.31] | 0.001* | 1.11 [0.81 – 1.50] | 0.518 | 2.02 [1.23 – 3.32] | 0.006* |
|  |  |  |  |  |  |  |
| *Trauma: neglect* | OR [95% CI] | P | OR [95% CI] | P | OR [95% CI] | P |
| Age | 0.99 [0.97 – 1.01] | 0.202 | 0.98 [0.97 – 1.00] | 0.003* | 0.99 [0.97 – 1.01] | 0.208 |
| Gender | 0.45 [0.32 – 0.63] | <0.001* | 0.82 [0.63 – 1.07] | 0.146 | 0.58 [0.38 – 0.88] | 0.011* |
| Neglect | 1.48 [1.08 – 2.02] | 0.014* | 1.15 [0.86 – 1.55] | 0.347 | 1.59 [0.98 – 2.57] | 0.062 |
| Corrected for age and gender. * p-value <0.017, considering Bonferroni correction for multiple testing.  Abbreviations: OR = Odds Ratio; [95% CI] = 95% confidence interval. | | | | | | |

**Supplement 7.** Associations between childhood trauma and numbers of cigarettes in the patients

| *Trauma* | *β* | *SE* | *p-value* |
| --- | --- | --- | --- |
| Total trauma | 2.33 | 0.85 | 0.006* |
| (Constant) | 15.22 | 1.96 | <0.001 |
|  |  |  |  |
| Trauma: abuse | 3.35 | 0.85 | <0.001* |
| (Constant) | 15.06 | 1.94 | <0.001 |
|  |  |  |  |
| Trauma: neglect | 1.21 | 0.86 | 0.160 |
| (Constant) | 15.50 | 1.96 | <0.001 |
| Corrected for age and gender (model 1). * p-value <0.017, considering Bonferroni correction for multiple testing. Abbreviations: SE=standard error. | | | |

**Supplement 8.** Associations between childhood trauma and tobacco smoking in patients and controls while corrected for multiple confounders (model 2)

|  | **Patients (N=610)** | | **Controls (N=435)** | |
| --- | --- | --- | --- | --- |
| *Total trauma* | OR [95% CI] | p | OR [95% CI] | p |
| Age | 0.99 [0.96 – 1.01] | 0.330 | 0.99 [0.97 – 1.01] | 0.446 |
| Gender | 0.56 [0.37 – 0.83] | 0.005* | 0.60 [0.36 – 0.98] | 0.040 |
| Education | 0.94 [0.90 – 0.99] | 0.018 | 1.01 [0.94 – 1.08] | 0.898 |
| CAPE positive | 1.45 [ 0.92 – 2.29] | 0.114 | 1.09 [0.17 – 6.82] | 0.927 |
| CAPE negative | 1.03 [0.64 – 1.65] | 0.906 | 0.58 [0.21 – 1.64] | 0.307 |
| CAPE depressive | 1.10 [ 0.67 – 1.80] | 0.709 | 2.32 [0.94 – 5.71] | 0.068 |
| Cannabis use | 16.41 [5.06 – 53.17] | <0.001* | 10.18 [2.69 – 38.45] | <0.001* |
| Total trauma | 1.32 [0.91 – 1.91] | 0.139 | 1.78 [1.00 – 3.14] | 0.048 |
|  |  |  |  |  |
| *Trauma: abuse* |  |  |  |  |
| Age | 0.99 [0.96 – 1.01] | 0.328 | 0.99 [0.97 – 1.02] | 0.543 |
| Gender | 0.55 [0.36 – 0.83] | 0.004* | 0.59 [0.36 – 0.97] | 0.037 |
| Education | 0.94 [0.90 – 0.99] | 0.016* | 1.00 [0.93 – 1.08] | 0.961 |
| CAPE positive | 1.41 [0.89 – 2.24] | 0.140 | 1.25 [0.20 – 7.81] | 0.809 |
| CAPE negative | 1.04 [0.65 – 1.66] | 0.888 | 0.60 [0.22 – 1.69] | 0.335 |
| CAPE depressive | 1.08 [0.66 – 1.78] | 0.757 | 2.46 [1.00 – 6.06] | 0.050 |
| Cannabis use | 16.47 [5.09 – 53.37] | <0.001* | 10.58 [2.81 – 39.85] | <0.001* |
| Abuse | 1.35 [0.92 – 1.98] | 0.128 | 1.41 [0.77 – 2.56] | 0.263 |
|  |  |  |  |  |
| *Trauma: neglect* |  |  |  |  |
| Age | 0.99 [0.97 – 1.01] | 0.374 | - | - |
| Gender | 0.56 [0.38 – 0.85] | 0.006* | - | - |
| Education | 0.94 [0.90 – 0.99] | 0.020 | - | - |
| CAPE positive | 1.49 [0.95 – 2.36] | 0.085 | - | - |
| CAPE negative | 1.02 [0.64 – 1.64] | 0.926 | - | - |
| CAPE depressive | 1.14 [0.70 – 1.86] | 0.609 | - | - |
| Cannabis use | 16.97 [5.24 – 54.95] | <0.001* | - | - |
| Neglect | 1.11 [0.77 – 1.60] | 0.574 | - | - |
| Corrected for age, gender, education, positive symptoms, negative symptoms, depressive symptoms, and cannabis use. * p-value <0.017, considering Bonferroni correction for multiple testing. Abbreviations: OR = Odds Ratio; [95% CI] = 95% confidence interval; CAPE = Community Assessment of Psychic Experience, frequency subscales. | | | | |

**Supplement 9.** Associations between childhood trauma and tobacco smoking in patients with additional covariates (antipsychotic medication use and PANSS scores)

| *Trauma (N=510)* | OR [95% CI] | p-value |
| --- | --- | --- |
| Total trauma | 1.58 [1.05 – 2.38] | 0.029 |
| Abuse | 1.53 [1.01 – 2.32] | 0.046 |
| Neglect | 1.36 [0.90 – 2.05] | 0.148 |
| Model 2: corrected for age, gender, education, positive symptoms, negative symptoms, general symptoms, cannabis use, and antipsychotic medication use. * p-value <0.017, considering Bonferroni correction for multiple testing. Abbreviations: OR = Odds Ratio; 95% CI = 95% confidence interval. | | |

**Supplement 10.** Associations between childhood trauma and tobacco smoking in patients with additional covariates (antipsychotic medication dosage, antipsychotic medication type, and PANSS scores)

| *Trauma (N=432)* | OR [95% CI] | p-value |
| --- | --- | --- |
| Total trauma | 1.47 [0.94 – 2.31] | 0.095 |
| Abuse | 1.48 [0.94 – 2.33] | 0.095 |
| Neglect | 1.22 [0.77 – 1.92] | 0.403 |
| Model 2: corrected for age, gender, education, positive symptoms, negative symptoms, general symptoms, cannabis use, antipsychotic medication dosage and antipsychotic medication type. * p-value <0.017, considering Bonferroni correction for multiple testing. Abbreviations: OR = Odds Ratio; 95% CI = 95% confidence interval. | | |

**Supplement 11.** Associations between childhood trauma and numbers of cigarettes in controls

| *Trauma* | *β* | *SE* | *p-value* |
| --- | --- | --- | --- |
| Total trauma | 2.67 | 0.67 | <0.001* |
| (Constant) | 4.74 | 1.17 | <0.001 |
| Corrected for age and gender (model 1). * p-value <0.017, considering Bonferroni correction for multiple testing. Abbreviations: SE=standard error. | | | |
